# Bacterial Diversity, Metabolic Profiling, and Application Potential of Antarctic Soil Metagenomes

**DOI:** 10.3390/cimb46110785

**Published:** 2024-11-18

**Authors:** Mario Fernández, Salvador Barahona, Fernando Gutierrez, Jennifer Alcaíno, Víctor Cifuentes, Marcelo Baeza

**Affiliations:** 1 Departamento de Ciencias Ecológicas, Facultad de Ciencias, Universidad de Chile, Las Palmeras 3425, Santiago 7800003, Chilefernagutierrez5@gmail.com (F.G.); jalcainog@uchile.cl (J.A.);; 2 Facultad de Ciencias, Universidad de Chile, Las Palmeras 3425, Santiago 7800003, Chile; salvador@uchile.cl

**Keywords:** shotgun metagenomes, Antarctica, bacterial diversity, metabolic profiling, applied enzymes

## Abstract

Antarctica has attracted increasing interest in understanding its microbial communities, metabolic potential, and as a source of microbial hydrolytic enzymes with industrial applications, for which advances in next-generation sequencing technologies have greatly facilitated the study of unculturable microorganisms. In this work, soils from seven sub-Antarctic islands and Union Glacier were studied using a whole-genome shotgun metagenomic approach. The main findings were that the microbial community at all sites was predominantly composed of the bacterial phyla Actinobacteria and Cyanobacteria, and the families Streptomycetaceae and Pseudonocardiaceae. Regarding the xenobiotic biodegradation and metabolism pathway, genes associated with benzoate, chloroalkane, chloroalkene, and styrene degradation were predominant. In addition, putative genes encoding industrial enzymes with predicted structural properties associated with improved activity at low temperatures were found, with catalases and malto-oligosyltrehalose trehalohydrolase being the most abundant. Overall, our results show similarities between soils from different Antarctic sites with respect to more abundant bacteria and metabolic pathways, especially at higher classification levels, regardless of their geographic location. Furthermore, our results strengthen the potential of Antarctic soils as a source of industrially relevant enzymes.

## 1. Introduction

The Antarctic continent has long been of interest to the scientific community due to its extreme environmental and geographic conditions, which make it one of the most inhospitable and isolated places on the planet. Most of the continent is covered by a permanent layer of ice and snow, but the 2% of ice-free regions encompass a variety of marine and terrestrial environments that support the vast majority of the continent’s biodiversity [1,2,3], with biomes with different biotic and abiotic factors hosting diverse microbial communities in their soils [3,4]. Initially, knowledge of microbial communities was based on culture-dependent studies in which most of the described bacteria belonged to the phyla Actinobacteria and Firmicutes and were represented by more than 70 genera, such as *Arthrobacter*, *Micrococcus*, *Cellulomonas*, *Rhodococcus*, and *Flavobacterium* [5,6]. In the case of yeasts, the top five genera described from these regions are *Mrakia*, *Naganishia*, *Rhodotorula*, *Candida*, and *Leucosporidium* [7]. It is well known that only a small fraction of environmental microorganisms can be cultured using current methods, especially those from extreme environments [8], and metagenomic approaches, driven by advances in next-generation sequencing (NGS) technologies, has emerged as a valuable tool for uncovering the intricate microbial diversity of the many microbiomes of Antarctica and other extreme environments [9,10,11]. Using these approaches, thousands of species belonging to the phyla Firmicutes, Proteobacteria, Flavobacteria, Sphingobacteria, Cytophaga, and Actinobacteria have been identified in different sites in Antarctica [12,13,14,15]. In general, fungal sequences are less abundant than bacterial sequences and have been detected more effectively when targeted by amplicon metagenomic sequencing via DNA metabarcoding in various studies [16,17,18,19,20,21]. Shotgun metagenomics, in addition to taxonomic description, allows the functional characterization of samples by identifying genes and mapping them to metabolic pathways, as the identification of genes related to the degradation and metabolism of aromatic and aliphatic hydrocarbons have been identified in soils from Deception and Livingston Islands [22].

Environmental microorganisms produce and secrete hydrolytic enzymes to utilize the carbon sources available in their environment. In the case of cold environments, these “cold-active” or “psychrophilic” enzymes have significant biotechnological potential in several industrial processes due to their ability to catalyze reactions at low temperatures [10,23,24,25,26]. Therefore, gene mining of soil metagenomes provides an opportunity to explore and exploit the vast genetic reservoir and metabolic capabilities inherent in soil microbiomes and infer functional potential and metabolic networks from genome-encoded enzymes [27,28,29].

In this work, whole-genome shotgun (WGS) metagenomic sequencing was used to explore the microbial diversity and metabolic potential in Antarctic soil samples from five islands in the South Shetland Archipelago, two islands near the Antarctic Peninsula, and Union Glacier. In addition, the presence of putative genes encoding enzymes of interest for application in productive areas and their predicted protein structural properties were investigated.

## 2. Materials and Methods

### 2.1. Sampling Sites

**Figure 1:**
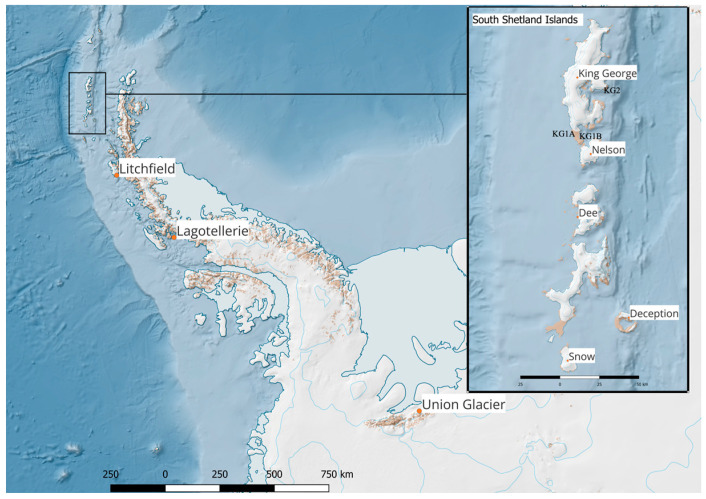
Locations of the islands of the Shetland South Archipelago and the Antarctic Peninsula, and Union Glacier. KG1A: King George Island Site 1A. KG1B: King George Island Site 1B, KG2: King George Island Site 2.

Soil samples from King George, Deception, Snow, Dee, Nelson, Litchfield, and Lagotellerie Islands and Union Glacier were collected during several expeditions to Antarctica as previously described [30,31,32,33] (Figure 1). These samples were placed in sterile 50 mL plastic tubes, sealed, and shipped at −20 °C to the laboratory of the Universidad de Chile in Santiago where they were stored at −80 °C until further processing. For convenience, the following site abbreviations were used in figures and tables: King George Island Site 1A (KG1A), King George Island Site 1B (KG1B), King George Island Site 2 (KG2), Deception Island (Decep), Snow Island (Snow), Dee Island (Dee), Nelson Island (Nelson), Litchfield Island (Litch), Lagotellerie Island (Lago), and Union Glacier (UG).

### 2.2. DNA Extraction, Library Preparation, and Sequencing

DNA was extracted from soil samples from sites collected from nine islands in the subantarctic region and Union Glacier (Table S1) using the PowerSoil^®^ DNA Isolation Kit (MO BIO Laboratories Inc., Carlsbad, CA, USA) according to the manufacturer’s instructions, except for the disruption step, which was performed using a Mini-Beadbeater-16 cell disruptor (BioSpec, Bartlesville, OK, USA) instead of vortex agitation. The extracted DNA was quantified using a Qubit 4 fluorometer (Invitrogen, Waltham, MA, USA), and those that reached the quality and concentration required for NGS were selected and pooled according to the corresponding island (Table S1) and sent to Omics2View consulting (Kiel, Germany) for processing, sequencing, and informatics data analysis. Short insert shotgun sequencing libraries were prepared by shearing the DNA by sonication and agarose gel electrophoresis to retain fragments of the desired length. P5/P7 Illumina adapters and 8-nt index sequences were ligated to the DNA fragments. Pair-end (2 × 150 bp) Illumina HiSeq X Ten sequencing was performed. Data were submitted to the National Center for Biotechnology Information, Bioproject PRJNA1038696.

### 2.3. Bioinformatic Processing and Data Analysis

The quality of the demultiplexed reads was evaluated using FastQC v0.11.7 [34]. Read quality trimming was performed using the BBTools v38.45 package [35], which included removal of optical duplicates, human sequences, adapter sequences, low entropy reads, and trimming of bases with quality scores < 10, as well as reads with invalid or ambiguous bases and reads < 127 base pairs (bp) in length. Read quality recalibration and error correction were performed using the BBTools v38.45 package [35] and aligned to a preliminary de novo assembly using Tadpole from a subset of the quality-trimmed reads, with information used to recalibrate the base quality of all quality-trimmed reads. Sequence errors were corrected by applying BBMerge, Clumpify, and Tadpole in error correction mode to the quality-recalibrated reads, and 31-bp Kmer reads were normalized with BBNorm to a target Kmer depth of 100×, with a minimum Kmer depth of 3× (filtered reads). Cross-assemblies were constructed using MEGAHIT v1.1.4-2-gd1998a1 [36,37], run with a preset “meta-large” and a minimum contig length of 1000 bp, and the generated scaffolds were subjected to an additional scaffolding step using SSPACE standard v3.0 [38] with default parameters (primary assembly). Filtered reads were back-mapped to the primary assembly using BBMap from the BBTools v38.45 package [35], and overall coverage was determined from the number of unambiguously mapped reads for each primary assembly contig and contigs with length ≥ 1000 bp and coverage ≥ 10× were considered (filtered assembly). Assembly statistics were generated using code from the “Assemblathon 2” project [39] (Table S2). Read counts per sample were generated from alignments to filtered contigs using SAMtools v1.9 [40]. The length distribution of the generated contigs is shown in Figure S1.

### 2.4. Taxonomic Assignment

Taxonomic assignment of contigs was accomplished with Kraken v2.0.8 [41]. Genomic information of bacteria, archaea, viruses, protozoa, fungi, plasmids, and Homo sapiens as well as vector sequences retrieved on 28 February 2020 were used as references with corresponding taxonomic information. Low-complexity regions within reference sequences were masked using DustMasker v1.0.0 [42] from the BLAST+ package v2.9.0 [43,44]. Hierarchical taxonomic classifications of contigs were in compliance with NCBI taxonomy [45] at standard taxonomic levels.

### 2.5. Coding Sequence Prediction and Functional Annotation

The contig analysis was performed using Geneious Prime version 2023.2.1 [46], hereafter referred to as Geneious Prime for simplicity. To predict coding sequences (CDS), the Geneious Prime plugins were used, including Glimmer [47,48] with genetic code 11 for bacteria and Augustus [49,50] for eukaryotes, using the general training. In both cases, only CDS of at least 300 nt were considered for functional annotation using the KAAS-KEGG Automatic Annotation Server [51], using the default parameters and selecting the appropriate gene dataset for prokaryotes or eukaryotes as needed. The length distribution of the generated CDSs is shown in Figure S1. Predicted CDSs were translated using standard genetic code and compared against a local database constructed with sequences from 34 types of enzymes [52,53] and antifreeze and ice-binding proteins downloaded from the UniProt database (https://www.uniprot.org/, accessed on 10 March 2022; 98,153 sequences in total) using BlastP with the configuration “Bin in ‘has hit’ vs. ‘no hit’” in the Geneious Prime software. CDSs with hits were then compared with the Swiss-Prot database and annotated with a similarity threshold of ≥30% using the Blosum62 cost matrix. The putative CDSs encoding potential industrial enzymes were compared with the SWISS-MODEL server (https://swissmodel.expasy.org, accessed on 2 November 2022) and models were generated if a suitable orthologous template was found (coverage ≥50%, similarity ≥30%, Global Model Quality Estimate (GMQE) ≥0.8). The quality of the generated models was evaluated using VERIFY 3D [54,55] (https://saves.mbi.ucla.edu/, accessed on 10 December 2022) and those models that passed this evaluation were used to calculate parameters associated with protein structural flexibility, such as the number of hydrogen bonds, total solvent-accessible surface area (TotSAS), apolar solvent-accessible surface area (ApoSAS), number of salt bridges, flexibility, and secondary structures content [23,24,56,57]. The Chimera and ChimeraX-1.17 software [58,59] were used to calculate TotSAS, ApoSAS, and the number of hydrogen bonds and salt bridges using the default parameters. The following parameters were used to estimate TotSAS and ApoSAS: radius = 1.4 Å, peak density = 2. To estimate hydrogen bonds and salt bridges, the following settings were used: radius = 0.075 Å, dashes = 6, distance tolerance = 0.4 Å, angle tolerance = 20°. Secondary structure content was extracted from the PDB files using the 2struccompare metaserver (https://2struccompare.cryst.bbk.ac.uk/index.php, accessed on 15 January 2023). Protein flexibility was calculated using MEDUSA [60] as the percentage of residues in regions predicted to be rigid (0), flexible (1), and very flexible (2).

### 2.6. Statistical Analyses and Data Processing

The Lilliefors, Kolmogorov–Smirnov, Anderson–Darling, and D‘Agostino’s K-squared tests rejected the normality of the data. Statistical comparisons were made using the Kruskal–Wallis test, followed by Dunn’s post hoc test (*p* < 0.05). Statistical analysis, Shannon index, and Bray–Curtis dissimilarity calculations were conducted in Python using the pandas and SciPy packages [61,62]. Graphs were generated using the Matplotlib, NumPy, and scikit-learn packages [63,64].

## 3. Results

### 3.1. Analysis of Bacterial Taxa in Soils from Different Antarctic Sites

**Figure 2:**
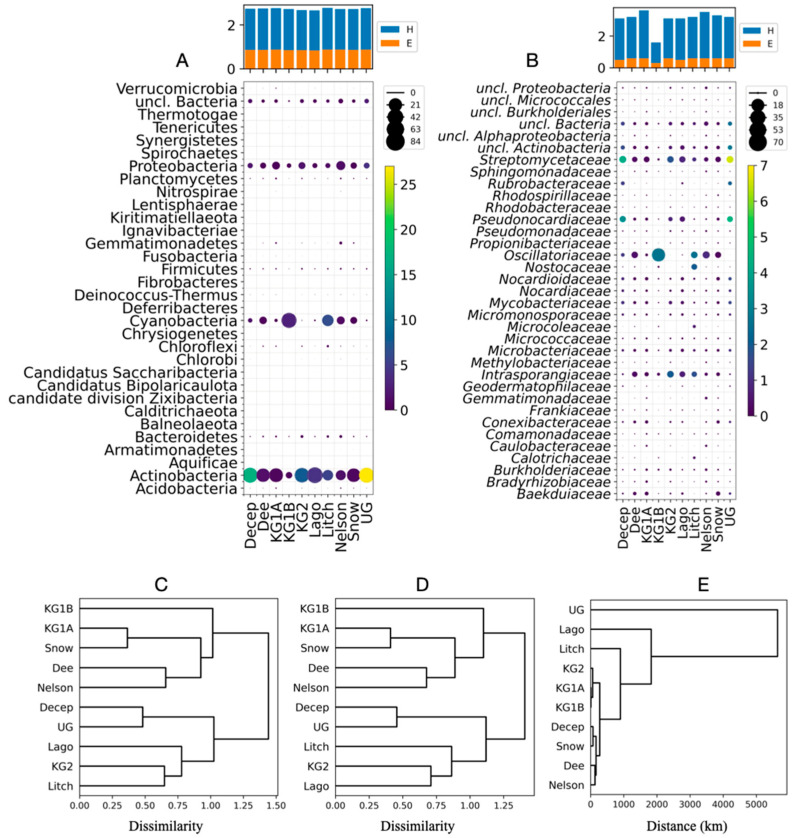
Distribution and abundance of different bacterial taxa at Antarctic sites. The percentage of each taxon classified by Kraken was calculated at each site (circle size) and relative to all sites (circle color), at the level of phyla (**A**) and families (**B**), with only the 35 most abundant taxa shown. The calculated Shannon index (H) and evenness (E) are shown in the bar plots. Hierarchical clustering of sites according to the Bray–Curtis dissimilarity values calculated for bacterial phyla (**C**), bacterial family (**D**), and distance in kilometers between sites (**E**).

Taxonomic analysis was performed on bacteria as the reads associated with this domain represented almost all reads in each studied site (99.5% to 99.9%). A total of 33 bacterial phyla were identified, 20 of which were detected at all 10 sites (Figure 2A). Actinobacteria was the most abundant phylum, accounting for 66% of all data, and was the most abundant phylum at eight sites, with higher percentages at Union Glacier (27%) and Deception Island (17%). Cyanobacteria and Proteobacteria were the second and third most abundant phyla, representing 13% and 12% of all data, respectively. Cyanobacteria was the predominant phylum at King George Island Site 1B (77%) and Lichfield Island (45%). The remaining phyla represent small percentages by location (lower 3%). A total of 320 bacterial families were found, 126 of which were identified at all sites (Figure 2B, Table S3). Five families represented 45% of all reads associated with bacteria: Streptomycetaceae, Pseudonocardiaceae, Oscillatoriaceae, uncl. Actinobacteria, and Intrasporangiaceae. By site, the most abundant families were Streptomycetaceae at Deception, King George Island Site 1A, and Lagotellerie Islands and Union Glacier, Oscillatoriaceae at Dee, King George Island Site 1B (which was particularly high at this site, 70%), Litchfield, Nelson, and Snow Islands, and Intrasporangiaceae at King George Island Site 2.

According to the calculated Shannon index (H), the biodiversity of bacterial phyla was similar at all sites, ranging from 2.7 to 2.8, with evenness (E) values ranging from 0.8 to 0.9 (Figure 2A). Similar results were obtained for bacterial families (H values ranging from 3.1 to 3.6 and E values from 0.5 to 0.6), with the exception of King George Island Site 1B where lower indices were obtained (H = 1.6, E = 0.3) (Figure 2B). Variability among sites was assessed by calculating Bray–Curtis dissimilarity for bacterial phyla and families, which was used for hierarchical clustering of sites, generating identical dendrograms at the two taxonomic levels (Figure 2C,D). The way in which sites were grouped according to taxonomic data differs from hierarchical clustering based on geographic distance between sites (Figure 2E). For example, Deception Island, geographically closer to Snow, Dee, and Nelson Islands, was grouped with Union Glacier, the most distant site from the others. King George Island sites were grouped separately with other islands, King George Site 1A with Snow Island, and King George Site 2 with the more distant Lagotellerie Islands.

### 3.2. Metabolic Potential of Soils from Different Antarctic Sites

**Figure 3:**
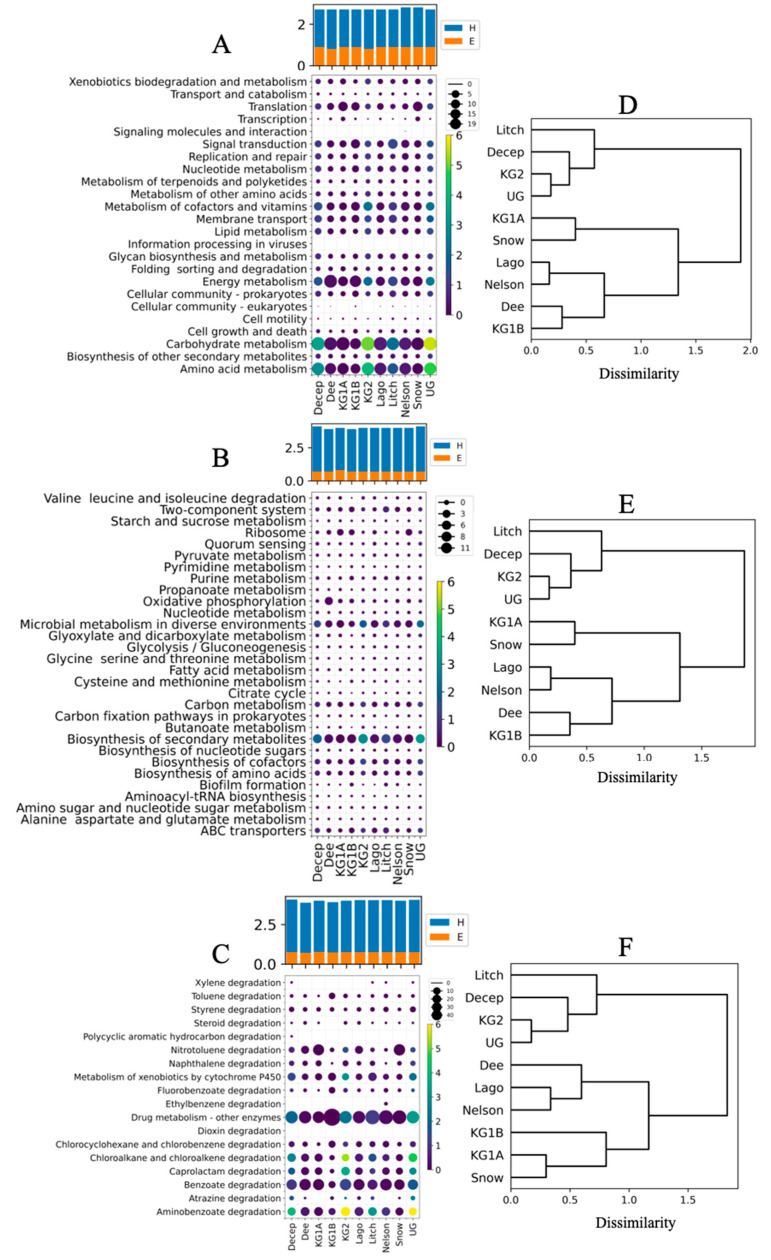
Metabolic characterization of the Antarctic sites. The percentage of each pathway or bacterial genus was calculated at each site (circle size) and relative to all sites (circle color). (**A**) Intermediate level pathways, (**B**) specific level pathways (only the 30 most abundant are shown), (**C**) xenobiotic biodegradation and metabolism pathways. The calculated Shannon index (H) and evenness (E) are shown in the bar plots. Hierarchical clustering of sites according to the Bray–Curtis dissimilarity values calculated for Intermediate level pathways (**D**), specific level pathways (**E**), xenobiotic biodegradation and metabolism pathways (**F**).

A total of 29% (40,780) of all predicted CDSs in the metagenomes were annotated and classified into general, intermediate, and specific pathways according to the Kyoto Encyclopedia of Genes and Genomes. The reads were associated with 24 intermediate metabolic pathways across all sites. Ten of these pathways accounted for 77% of all reads, and the most represented were carbohydrate metabolism, amino acid metabolism, and energy metabolism, with 41% of the total (Figure 3A). These three pathways were more abundant at Deception, King George Site 2, Litchfield Islands, and Union Glacier. A total of 233 specific metabolic pathways were found, 186 were detected in all sites, and 30 of them account for 62% of the assigned reads (Figure 3B and Table S4). Biosynthesis of secondary metabolites and microbial metabolism in diverse environments consistently were the first and second most abundant metabolic pathways in the studied sites, except for Dee Island, where the second most abundant was oxidative phosphorylation. Thirteen of the 18 specific pathways of the category “xenobiotic biodegradation and metabolism” were identified at all sites, whereas dioxin degradation was identified at only five sites (Figure 3C). 80% of all reads were associated with seven pathways, and the “drug metabolism—other enzymes” pathway was consistently the most abundant at all sites (10% to 40%). The second most abundant pathway was benzoate degradation in six islands and Union Glacier (14% to 19%), nitrotoluene degradation at King George site 1A (17%) and Snow Island (18%), and metabolism of xenobiotics by cytochrome P450 at King George site 1B (9%). The calculated Shannon and evenness indices for each metabolic level were similar among sites, with H ranging from 2.7 to 2.8 and E from 0.8 to 0.9 at the intermediate level, H ranging from 3.9 to 4.1 and E from 0.7 to 0.8 at the specific level, and H ranging from 2.1 to 2.4 and E from 0.7 to 0.8 for xenobiotic biodegradation and metabolism (Figure 3A–C). Hierarchical clustering of sites based on calculated Bray–Curtis dissimilarity yielded identical dendrograms for intermediate and specific pathways (Figure 3D,E), which differed from the dendrogram generated by “xenobiotic biodegradation and metabolism” (Figure 3F). In the first two, Dee Island and King George Island site 1B were closely grouped, but in the third case, Dee was grouped with Lagotellerie and Nelson Island, and George Island site 1B was grouped with George Island site 1A and Snow Island. Similar to the taxonomic analysis, this clustering is inconsistent with the geographic distances of the sites studied.

### 3.3. Gene Mining for Putative Enzymes with Potential Applications and Protein Structure Analysis

**Figure 4:**
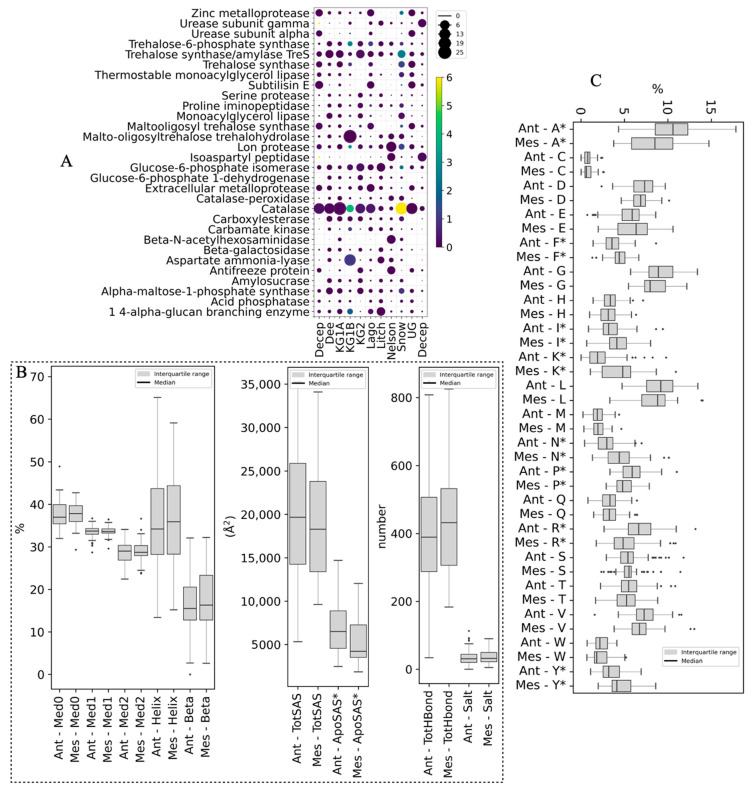
Enzymes with potential industrial applications and structural comparisons. (**A**) Percentage of CDS potentially encoding each enzyme at each site (circle size) and relative to all sites (circle color). (**B**) Distribution of calculated values for structural parameters from translated and modeled putative enzymes: alpha helix (Helix), beta strand (Beta), unstructured (O), residues classified as rigid (Med0), residues classified as flexible (Med1), residues classified as very flexible (Med2), total solvent-accessible surface area (TotSAS), apolar solvent-accessible surface area (ApoSAS), total hydrogen bonds (TotHbond), and salt bridges (Salt). (**C**) Distribution of amino acid percentages (one-letter code). Ant: putative enzymes found in metagenomes in this work and Mes: mesophilic enzyme orthologs. Significant differences between Ant and Mes were found in the parameters indicated with *.

A total of 1244 CDSs encoding 71 types of enzymes with potential industrial applications were predicted in all metagenomes (Figure 4A). Twenty-eight putative enzymes were found in all sites, and 17 of them accounted for 70% of all associated reads. The percentage of reads associated with catalase enzymes was highest when data from all sites were combined, and was highest at six islands and Union Glacier. King George Island Site 1B, Nelson, and Litchfield Islands had the highest percentages of reads associated with malto-oligosyltrehalose trehalohydrolase, glucose-6-phosphate isomerase, and 1,4-alpha-glucan branching enzyme. The top 10 putative enzymes with higher percentages considering all site data were, in addition to those mentioned above, trehalose synthase/amylase TreS, Lon protease, aspartate ammonia-lyase, alpha-maltose-1-phosphate synthase, extracellular metalloprotease, and trehalose synthase. CDSs encoding the antifreeze proteins were found at all sites except King George Site 1B, with higher percentages at Nelson and Deception Islands and Union Glacier.

To evaluate the putative enzymes for structural flexibility, the CDSs were translated in silico, compared with the SWISS-MODEL database, and 3D models were constructed and quality-checked when a suitable ortholog was found. In this way, 87 protein models were generated and the parameters proposed as key factors for improved performance of cold-adapted enzymes at low temperatures, such as ionic-electrostatic interactions, solvent-accessible surface, hydrogen bonds, salt bridges, and the proportion of large amino acids, were calculated and compared with those calculated from the mesophilic ortholog. In the box plots of Figure 4B, some tendencies can be observed between the parameter values obtained from the Antarctic putative enzymes and their corresponding mesophilic orthologs. Antarctic enzymes tended to have a lower content of secondary structures, amino acids classified as rigid (by Medusa), and hydrogen bonds than mesophilic orthologs. The solvent-accessible surface area showed interesting results with a tendency for a higher total surface area for putative Antarctic enzymes than for mesophilic ones, which was significantly (*p* ≤ 0.05) higher when considering the apolar surface area. Regarding the percentage of amino acids, significant differences between Antarctic and mesophilic enzymes were found for eight amino acids (Figure 4C). A higher percentage of A, P, and R were found in the Antarctic than in mesophilic enzymes, residues that are classified as small or very small, with the exception of R. On the other hand, the Antarctic enzymes had lower percentages of F, I, K, N, and Y than the mesophilic enzymes, residues that, with the exception of N, are classified as large or very large.

## 4. Discussion

The present study investigated the bacterial and metabolic diversity and potential as a source of industrial enzymes in the metagenomes of soils from five islands in the Shetland South Archipelago, two from the Antarctic Peninsula, and Union Glacier, Antarctica. The most abundant bacterial phyla were Actinobacteria, Cyanobacteria, Proteobacteria, uncl. Bacteria, and Bacteroidetes in most of the study sites. These results are consistent with previous studies in soils from different Antarctic regions that reported Proteobacteria, Actinobacteria, and Bacteroidetes as the most abundant phyla in soils from King George and Livingston Islands [65], Proteobacteria and Actinobacteria as the dominant phyla in soils from Mars Oasis on Alexander Island [13], and Cyanobacteria, Actinobacteria and Proteobacteria in biological soil crusts in regions of Enderby Land and Queen Maud Land (East Antarctica) [66]. In a shotgun metagenomic study of soils from Barton Peninsula (King George Island), Proteobacteria, Bacteroidetes, Acidobacteria (10th in this work), Chloroflexi (seventh in this work), Planctomycetes (eighth in this work), and Actinobacteria were reported as the predominant phyla [67]. At the bacterial family level, 320 families were identified, 126 of which were ubiquitous across all sites examined. Nearly half of the reads associated with bacterial families were represented by five families, the three most abundant being Streptomycetaceae, Pseudonocardiaceae, and Oscillatoriaceae. Considering the top 10 bacterial families described in this work, seven of them have been described in previous metagenomic studies but with different abundances, the closest being Pseudonocardiaceae, Oscillatoriaceae, and Nocardioidaceae [68,69]. According to the Shannon and evenness indices, bacterial diversity at the phylum and family levels was similar among the sites studied here, except for King George Island site 1B, where lower values were calculated for bacterial families. This was reflected in the hierarchical clustering of the sites according to the Bray–Curtis dissimilarity calculated considering the abundance of the bacterial taxa, in which the sites were grouped in a way that was independent of their geographical location and the distance between them. Therefore, it can be concluded that bacterial diversity among the islands of the Shetland South Archipelago, the Antarctic Peninsula, and the Union Glacier sites studied here is rather homogeneous for the more abundant taxa, with differences in the less abundant taxa.

In terms of metabolic pathways, some homogeneity was observed across sites, with 24 intermediate and 186 specific pathways found at all, with variation in their abundance in each site. The most abundant intermediate pathways were carbohydrate metabolism, amino acid metabolism, and energy metabolism, and the most abundant specific pathways were biosynthesis of secondary metabolites, microbial metabolism in diverse environments, and biosynthesis of cofactors. Although the hierarchical clustering of sites by Bray-Curtis dissimilarity differed from that obtained using taxonomic data, in both cases sites were grouped independently of their geographic location. Interestingly, reads associated with 13 metabolic pathways of xenobiotic biodegradation and metabolism were identified at all sites. The three most abundant pathways considering all sites studied were drug metabolism–other enzymes, benzoate degradation, and aminobenzoate degradation. As observed in the taxonomic analysis, similar Shannon and evenness indices were obtained at all sites for all metabolic levels analyzed. Previously, 125 catabolic genes associated with the degradation and metabolism of aromatic and aliphatic hydrocarbons were reported from Deception and Livingston Islands, and the authors suggested that the genetic potential for hydrocarbon degradation was due to the natural occurrence of hydrocarbons [22]. Another possibility is the existence of compounds classified as “natural xenobiotics” produced by some microorganisms and plants in the environment [70,71], but, to the best of our knowledge, there are no reports on this topic in Antarctic environments. However, thanks to the development of sophisticated instrumentation, a class of xenobiotics called persistent organic pollutants (POPs) has been detected in Antarctic terrestrial and marine ecosystems from King George Island to McMurdo and Victoria Land [72,73,74].

Mining metagenomes for genes encoding enzymes with high activity at low temperatures and other properties suitable for industrial applications is a promising strategy for the heterologous production of novel enzymes in productive organisms such as the yeast *Saccharomyces cerevisiae*. Of the 71 hydrolytic enzymes with applied potential detected in this study, 28 were found at all sites. In previous studies of soil metagenomes from Barton Peninsula (King George Island), 162 putative genes encoding carbohydrate-active enzymes (CAZy), including candidates for lignocellulolytic enzymes [67], and genes potentially encoding cold shock and antifreeze proteins were predicted in Enderby Land and Queen Maud biocrusts [66]. In a metagenomic library constructed in *E. coli* using soil samples from the Pointe Géologie archipelago (Ile des Petrels), Terre Adélie, recombinant clones with lipase/esterase, amylase, protease, and cellulase activities were obtained [75]. In terms of the most predicted enzymes in this study, catalase is one of the most important enzymatic mechanisms of antioxidant defense [76,77] and has been widely described in microorganisms, including some from Antarctica [78,79], with applications in bioremediation as an indicator of hydrocarbon degradation in soil and removal of H_2_O_2_ from bleaching industry effluents [80]. The enzyme malto-oligosyltrehalose trehalohydrolase functions in the synthesis of trehalose, a compound involved in the response to abiotic stress [81], and is used in the biotechnological production of trehalose [82]. The 1,4-alpha-glucan branching enzyme is ubiquitous in different types of organisms and modifies the structure of starch and glycogen with several applications, including glycogen production [83].

In addition to finding putative genes encoding enzymes of interest, predicting enzyme performance at low temperatures is desirable because several putative genes encoding a particular enzyme may be found in a metagenome. Several factors have been described to improve the performance of cold-adapted enzymes at low temperatures, related to the structural flexibility of the protein [23,24,56,57]. Among these factors, the putative Antarctic enzymes studied here tended to have a lower content of secondary structures, amino acids classified as rigid (by Medusa), hydrogen bonds, residues classified as large or very large, and a higher content of residues classified as small or very small. The more distinctive parameter was the apolar solvent-accessible surface area, which was significantly higher for putative Antarctic enzymes than for mesophilic counterparts. Therefore, determining these parameters provides relevant information for selecting putative enzymes with greater structural flexibility.

## 5. Conclusions

The present metagenomic study shows the similarity of soils from different Antarctic islands and the Union Glacier with respect to the most abundant bacterial taxa, represented by the phyla Actinobacteria, Cyanobacteria, Proteobacteria, and the families Streptomycetaceae, Pseudonocardiaceae, and Oscillatoriaceae. The most abundant specific pathways identified were biosynthesis of secondary metabolites, microbial metabolism in diverse environments, and biosynthesis of cofactors. The potential of Antarctic soils as a source of genes encoding industrially relevant enzymes was confirmed. Furthermore, predictive parameters for selecting putative enzymes with better performance at lower temperatures, such as lower content of secondary structures, hydrogen bonds, and higher apolar solvent-accessible surface area, were proposed. Heterologous expression and characterization of putative enzymes will allow the confirmation of these predictive parameters and strengthen the potential of the proposed enzymes to be applied in processes performed at cold temperatures.

## Data Availability

RNA-seq data: National Center for Biotechnology Information, Bioproject: PRJNA1038696.

## Supplementary Materials

The following supporting information can be downloaded at: https://www.mdpi.com/article/10.3390/cimb46110785/s1, Figure S1. Distribution of the length in nucleotides of contigs (A) and predicted CDSs (B) in the samples from the Antarctic sites studied; Table S1: Soil samples used for DNA extraction by each site; Table S2: Assembly statistics; Table S3: Reads associated with bacterial families in each site; Table S4: Reads associated to specific metabolic pathways in each site.
