# Supplementary figures and images for: Bacterial Diversity, Metabolic Profiling, and Application Potential of Antarctic Soil Metagenomes
Source: Curr Issues Mol Biol. 2024 Nov 18;46(11):13165–78. doi: 10.3390/cimb46110785 (PMC11593224; doi:10.3390/cimb46110785)

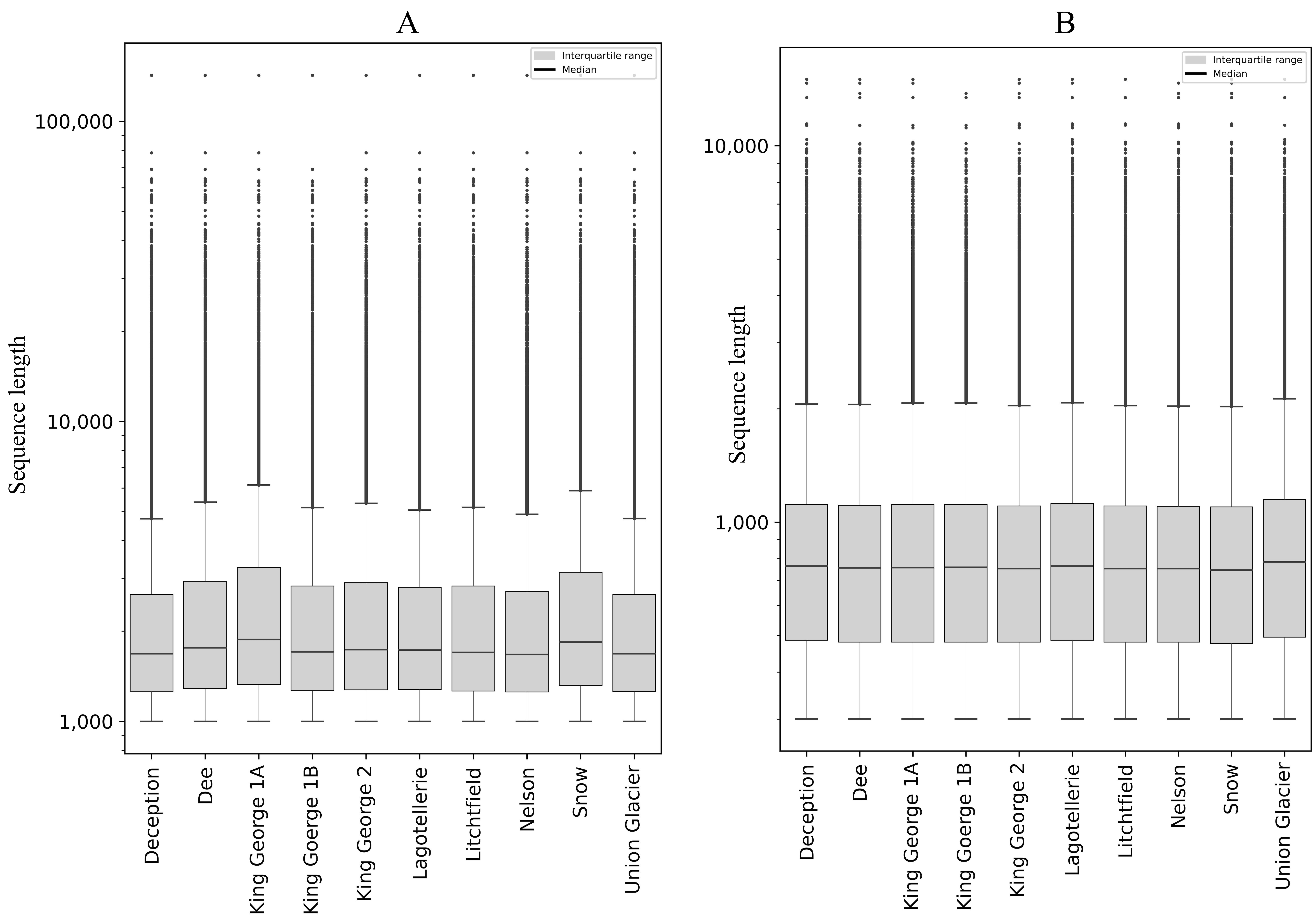

Supplement: Supplementary file 1 [file cimb-46-00785-s001.zip › cimb-3128480-figS1.jpg]
